# Comparative Analysis of Microbiome in Nasopharynx and Middle Ear in Young Children With Acute Otitis Media

**DOI:** 10.3389/fgene.2019.01176

**Published:** 2019-11-19

**Authors:** Qingfu Xu, Steve Gill, Lei Xu, Eduardo Gonzalez, Michael E. Pichichero

**Affiliations:** ^1^Center for Infectious Disease and Immunology, Rochester General Hospital Research Institute, Rochester, NY, United States; ^2^Department of Microbiology and Immunology, University of Rochester Medical Center, Rochester, NY, United States

**Keywords:** nasopharyngeal microbiome, middle ear microbiome, acute otitis media, 16S rRNA, Shannon Diversity, sample storage

## Abstract

Acute otitis media (AOM) is the most common pediatric infection for which antibiotics are prescribed in the United States. The role of the respiratory tract microbiome in pathogenesis and immune modulation of AOM remains unexplored. We sought to compare the nasopharyngeal (NP) microbiome of children 1 to 3 weeks prior to onset of AOM vs. at onset of AOM, and the NP microbiome with the microbiome in middle ear (ME). Six children age 6 to 24 months old were studied. Nasal washes (NW) were collected at healthy visits 1 to 3 weeks prior to AOM and at onset of AOM. The middle ear fluids (MEF) were collected by tympanocentesis at onset of AOM. Samples were stored in Trizol reagents or phosphate-buffered saline (PBS) at −80°C until use. The microbiome was characterized by 16S rRNA gene sequencing. Taxonomic designations and relative abundance of bacteria were determined using the RDP classifier tool through QIIME. Cumulative sum scaling normalization was applied before determining bacterial diversity and abundance. Shannon diversity index was calculated in Microsoft excel. The relative abundance of each bacteria species was compared *via* Mann-Whitney *U* test. We found that the NW microbiome of children during healthy state or at baseline was more diverse than microbiome during AOM. At AOM, no significant difference in microbiome diversity was found between NW and MEF, although some bacteria species appear to differ in MEF than in NW. The microbiome of samples stored in PBS had significant greater diversity than samples stored in Trizol reagent.

## Introduction

Acute otitis media (AOM) is one of the most common bacterial infections in children for which antibiotics are prescribed in the United States of America (Vergison et al., 2010; Monasta et al., 2012). The three major bacterial pathogens *Streptococcus pneumoniae*, *Haemophilus influenzae*, and *Moraxella catarrhalis* are among hundreds species of commensal microbiomes in the respiratory tract. Current prevention and treatment options are being continuously eroded by emergence of new otopathogen strains (Pettigrew et al., 2012). It is estimated that each year, more than 5 million AOM cases occur in the US (Monasta et al., 2012; Suaya et al., 2018). The annual total cost is about $6 billion in the US for health care of OM including $3 billion to 4 billion in direct costs for treatment of OM, and the most frequent surgery in children (after circumcision) involving insertion of tympanostomy tubes (Nsouli 2019).

Nasopharyngeal (NP) colonization by potential bacterial respiratory pathogens is a frequent event in early childhood, and initial, necessary step in pathogenesis of respiratory bacterial infectious diseases such as AOM, conjunctivitis, sinusitis, chronic obstructive pulmonary disease, and pneumonia in children (Bogaert et al., 2004; Libson et al., 2005; Syrjanen et al., 2005; Revai et al., 2008; Littman and Pamer 2011; Chonmaitree et al., 2017). Previous studies have shown that the microbiome plays an important role in modulating immune homeostasis and disease susceptibility (Duan et al., 2003; Kuss et al., 2011; Littman and Pamer 2011). A large portion of this research has focused on the gut microbiome and susceptibility to enteric pathogens (Sekirov et al., 2008; Kuss et al., 2011). The role of the respiratory tract microbiome in pathogenesis and immune modulation of AOM remain unexplored. Middle ear (ME) microbiome has been reported in chronic otitis media (Santos-Cortez et al., 2016; Krueger et al., 2017; Boers et al., 2018; Johnston et al., 2019), and NP microbiome is associated with pathogenesis of upper respiratory traction infection and AOM (Lappan et al., 2018). None of these studies investigate changes in NP microbiota during onset of AOM. Here we sought to compare the microbiome in nasal wash (NW) of children 2 to 3 weeks prior to their onset of AOM (but otherwise healthy) versus those same children at onset of AOM, and compare their NW microbiome with ME microbiome during AOM.

## Materials and Methods

### Study Cohorts and Samples

The NW and middle ear fluid (MEF) samples were previously collected under a US National Institutes of Health-funded study of AOM. The study design and sample collections have been described in previous publications (Xu et al., 2012; Pichichero 2016). Briefly, healthy infants without previous episodes of AOM were enrolled at 6 months of age and NW samples were prospectively collected at 6, 9, 12, 15, 18, 24, and 30 to 36 months of age. Whenever the children were diagnosed with AOM, tympanocentesis was performed on the same day. MEF samples were handled aseptically and kept on ice during transport to the lab, where it was processed immediately to confirm the diagnosis with microbiologic culture for otopathogens. The study was approved by the Institutional Review Board of Rochester General Hospital, and written informed consent was obtained from parents or guardians of all children. Samples were either directly stored in 1 ml phosphate-buffered saline (PBS) at −80°C, or centrifuged at 3,000 rpm for 10 min at 4°C, after which the pellets were stored in 1 ml of Trizol reagents (Sigma) at −80°C until use for microbiome analyses.

#### 16S rRNA Gene Sequencing Analysis

*Bacteria DNA Extraction*: Bacterial ribonucleotides from NP and MEF were extracted by FastPrep bead beating lysis in TRI Reagent (Ambion) and purified on a Zymo-Spin^™^ IC column (Zymo). Integrity of the purified nucleotides was assessed on an Agilent BioAnalyzer. The V1–V3 region of bacterial 16S rRNA genes were amplified using dual-indexed coded primers (Fadrosh et al., 2014) and Phusion High-Fidelity Polymerase (Thermo Fisher). V1–V3 amplicons were purified and normalized using SequalPrep^™^ Normalization plates (Life Technologies), pooled, and validated on an Agilent BioAnalyzer. The final library was paired-end sequenced (2 × 300 bp) on an Illumina MiSeq. The individual amplicons were pooled for sequencing. This approach routinely yields high quality sequence data, with ∼40 K reads per sample and assembly of 550 bp overlapping amplicons from the paired-end reads for each sample. This depth of sequencing coverage results in a high likelihood of identifying rare taxa.

To minimize the variations from sample processing, both DNA extraction and 16S rRNA gene sequencing analysis were simultaneously performed for all the samples.

*Processing of 16S rRNA Sequence Reads*: Raw data in the form of BCL files were processed into 2x300 FASTQ format paired end read files using Illumina's bcl2fastq version 1.8.4 without demultiplexing and with the EAMMS algorithm disabled. After preprocessing, the open source software package, Quantitative Insights Into Microbial Ecology (QIIME) (Caporaso et al., 2010), was used to remove low quality sequences and chimeras and to perform bacterial community quantification, description, and analyses. Specifically, assembled 16S rRNA reads were truncated at the beginning of the first 30 base window with a mean Phred quality score of less than 20 or at the first ambiguous base, whichever came first. Sequences were aligned and then processed by complete linkage clustering using a maximum cluster distance cutoff of 3% (97% identity) to define operational taxonomic units (OTUs). These OTUs were used to calculate Shannon and evenness diversity indices (Lozupone et al., 2007; Lozupone and Knight 2008).

*Taxonomic Description of the Respiratory Microbiome*: Taxonomic designations of our sequences was done using the RDP classifier tool, which uses a naive Bayesian method for taxonomic assignment and can be accessed through QIIME (Wang et al., 2007; Caporaso et al., 2010). The taxonomic OTU proportion was used to describe the NP and ME microbiomes within our population. Rank abundance plots were made listing the most frequent taxonomic OTU's within NP and ME samples. We note OTUs that are present in a given stratum but absent or at low levels in other strata. The differences in the abundance of individual taxa of interest between samples grouped by outcomes were analyzed by Mann-Whitney *U* test.

### Statistical Analysis

Shannon diversity index was calculated in Microsoft excel based on the equation $H\;=\;–\sum_{i}^{n}pi\;ln\left(pi\right)$ . which *p*_i_ is the portion of species i among the total population of n species in a sample. To minimize the discrepancy in data collection, only data from samples processed in Trizol were included in the comparison between healthy and AOM patients, in which three samples with the lowest DNA reads were excluded. Paired one-tailed *t* test was performed to measure the statistical significance between Shannon indexes using the GraphPad Prism software. The relative abundance of taxa was compared between samples by Mann-Whitney *U* test.

## Results

### Comparison of NP Microbiome at Onset of AOM and During Health Prior to AOM

**Figure 1:**
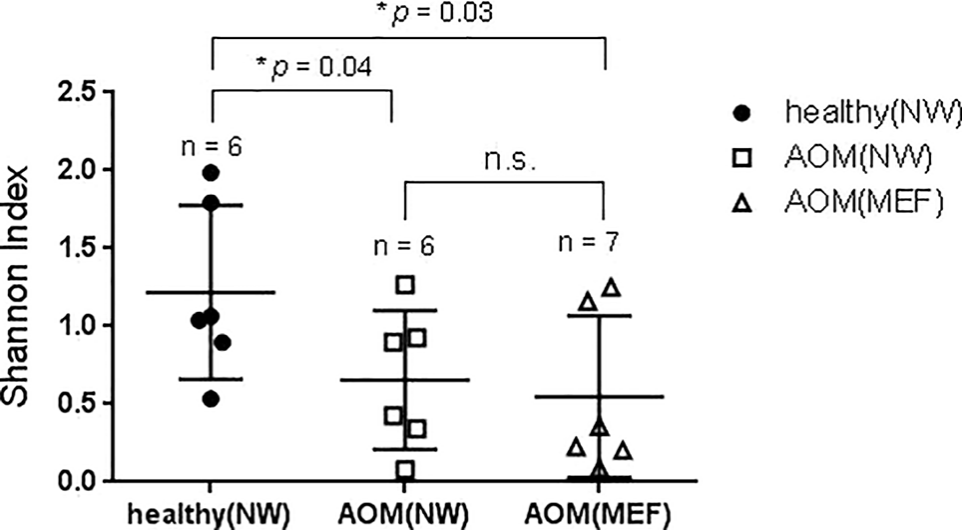
Diversity of NP and ME microbiome during health and AOM. The nasal wash (NW) and MEF samples were collected at onset of AOM and during heath prior to the AOM with 3 weeks' time interval. The samples were analyzed by 16S rRNA gene sequencing. Shannon diversity index was calculated and compared between samples (see *MATERIALS AND METHODS*) by one-tailed *t* test.

**Figure 2:**
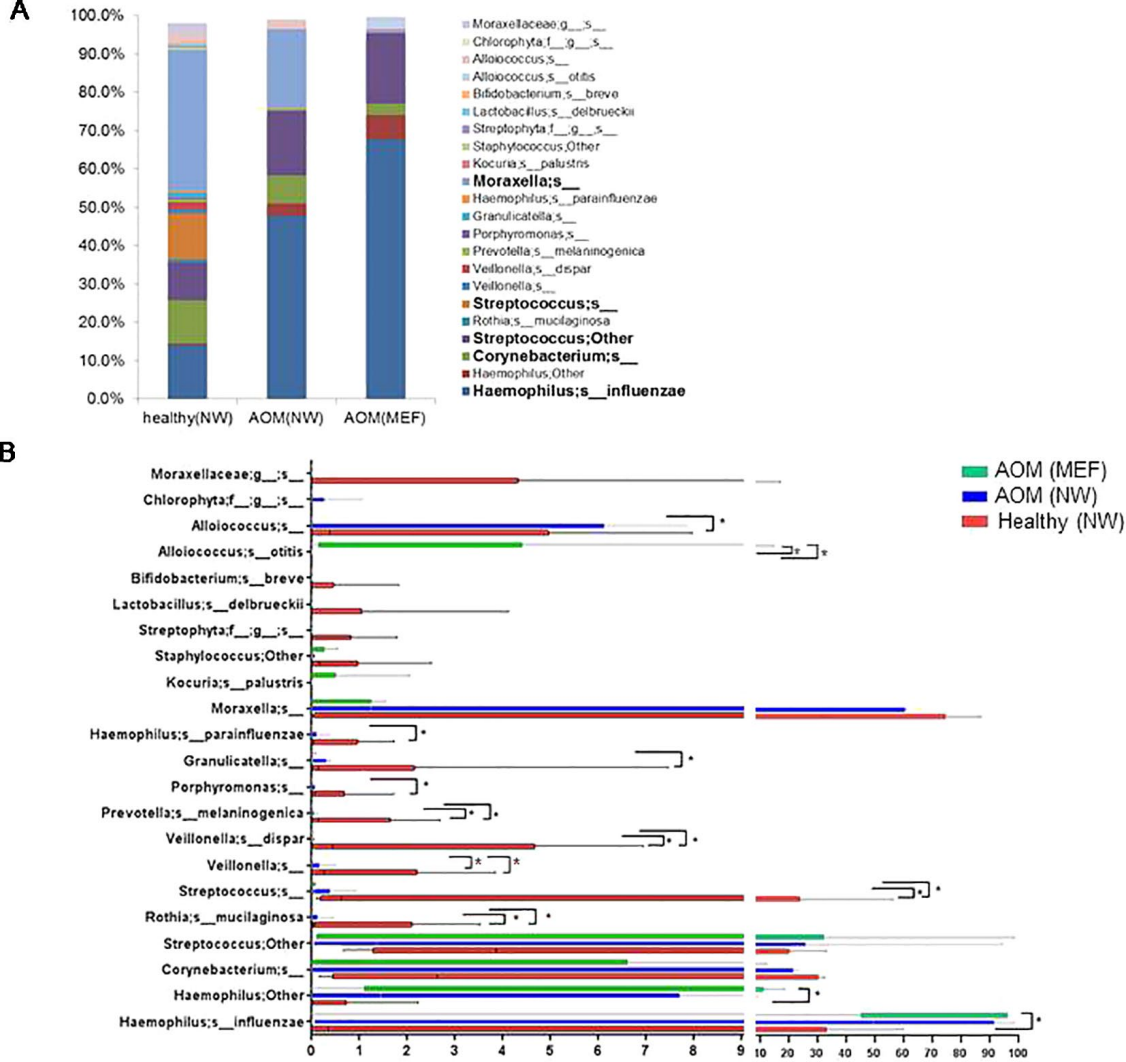
OTUs in NWs and MEFs during heath and at onset of AOM. The NW samples were collected at onset of AOM and during heath prior to the AOM with 3 weeks' time interval. The microbiome was analyzed by 16S rRNA gene sequencing and taxonomic designations. (A) Average abundance of OTUs in each group was plotted. (B) Comparison of abundance of each individual OUT between groups by Mann-Whitney test. *p < 0.05.

From our sample inventory, we identified six cases of children with a diagnosed AOM who happened to have a prospective healthy visit without any symptoms of URI or AOM at 1 to 3 weeks prior to AOM. This enabled us to perform a self-case-control analysis of NP microbiome during health vs. at onset of AOM. The results are summarized in the **Table 1** and **Figures 1** and **2**. We found that the NP microbiome had significantly greater diversity during health than at onset of AOM (**Figure 1**). The genus whose abundance was >1% were 6 ± 3 (mean ± SE) in the NW during health, 3 ± 1 in the NW during AOM, and 3 ± 2 in the MEF during AOM (**Figure 2A** and **Table 1**). The most abundant microbiome at genus level were *Moraxella* (36.89%), *Streptococcus* (21.65%), *Haemophilus* (14.16%), *Corynebacterium* (11.31%), *Veillonella* (2.97%), and *Alloiococcus* (2.12%) in NW of healthy children; *Haemophilus* (51.01%), *Moraxella* (20.69%), *Streptococcus* (16.75%), *Corynebacterium* (7.43%), and *Alloiococcus* (2.24%) in NW of AOM children; and *Haemophilus* (74.05%), *Streptococcus* (18.43%), *Corynebacterium* (3.02%), and *Alloiococcus* (2.91%) in MEF of AOM children (**Table 1**). Mann-Whitney test was performed to identify the OTUs that differ statistically significantly between the NWs during health and the NWs during AOM. They were found to be *Rothia mucilaginosa*, *Streptococcus* sp., *Veillonella dispar*, and *Prevotella melaninogenica* (**Figure 2B** and **Table 2**). On the other hand, more OTUs differed significantly between NWs during health and MEFs during AOM. They included *Haemophilus* sp., *R. mucilaginosa*, *Streptococcus* sp., *V. dispar*, *P. melaninogenica*, *Porphyromonas* sp., *Granulicatella* sp., and *Alloiococcus* sp. (**Figure 2B** and **Table 2**).

**Table 1:** OTUs with > 1% abundance in NW or MEF microbiome during health prior to an AOM (<3 weeks) and at onset of AOM.

NW during health prior to AOM		MEF at onset of AOM		MEF at onset of AOM	
Moraxella;s_	36.89%	Haemophilus;s_influenzae	47.80%	Haemophilus;s_influenzae	67.79%
Haemophilus;s_influenzae	14.16%	Moraxella;s_	20.69%	Streptococcus;Other	18.43%
Streptococcus;s_	11.85%	Streptococcus;Other	16.75%	Haemophilus;Other	6.26%
Corynebacterium;s_	11.31%	Corynebacterium;s_	7.43%	Corynebacterium;s_	3.02%
Streptococcus;Other	9,80%	Haemophilus;Other	3.21%	Alloiococcus;s_otitis	2.91%
Moraxellaceae;g_;s_	2.88%	Alloiococcus;s_	2.24%		
Alloiococcus;s_	2.12%				
Veillonella;s_–_dispar	1.96%				
Granulicatella;s_	1.36%				
Veillonella;s_	1.01%				

**Table 2:** Difference in OTUs Abundance % between Groups.

	NW		MEF	*Mann-Whitney Test*		
	health	AOM	AOM	heath vs AOM (NW)	health vs AOM (MEF)	AOM (NW) vs AOM (MEF)
Haemophilus;s_influenzae	14.16	47.80	67.79	not sig	*p* = 0.027	not sig
Haemophilus;Other	0.42	3.21	6.26	not sig	*p* = 0.033	not sig
Corynebacterium;s_	11.31	7.43	3.02	not sig	not sig	not sig
Streptococcus;Other	9.80	16.75	18.43	not sig	not sig	not sig
Rothia;s_–_mucilaginosa	0.88	0.08	0.00	*p* = 0.015	*p* = 0.0026	not sig
Streptococcus;s_	11.85	0.22	0.03	*p* = 0.015	*p* = 0.015	not sig
Veillonella;s_	1.01	0.10	0.00	not sig	*p* = 0.0066	*p* = 0.025
Veillonella;s_dispar	1.96	0.02	0.01	*p* = 0.033	*p* = 0.0042	not sig
Prevotella;s_melaninogenica	0.72	0.02	0.00	*p* = 0.015	*p* = 0.0042	not sig
Porphyromonas;s_	0.36	0.03	0.00	not sig	*p* = 0.023	not sig
Granulicatella;s_	1.36	0.11	0.02	not sig	*p* = 0.01	not sig
Haemophilus;s_parainfluenzae	0.42	0.07	0.00	not sig	*p* = 0.039	not sig
Moraxella;s_	36.89	20.69	0.53	not sig	not sig	not sig
Kocuria;s_palustris	0.00	0.00	0.34	not sig	not sig	not sig
Staphylococcus;Other	0.55	0.03	0.15	not sig	not sig	not sig
Streptophyta;f_;g_;s_	0.38	0.01	0.00	not sig	not sig	not sig
Lactobacillus;s_delbrueckii	0.69	0.00	0.00	not sig	not sig	not sig
Bifidobacterium;s_breve	0.30	0.00	0.00	not sig	not sig	not sig
Alloiococcus;s_otitis	0.00	0.00	2.91	not sig	*p* = 0.01	*p* = 0.025
Alloiococcus;s_	2.12	2.24	0.00	not sig	*p* = 0.033	not sig
Chlorophyta;f_;g_;s_	0.00	0.18	0.00	not sig	not sig	not sig
Moraxellaceae;g_;s_	2.88	0.00	0.00	not sig	not sig	not sig

### Comparison of NP and Middle Ear Microbiome at Onset of AOM

We also compared NP microbiome and MEF microbiome in children at onset of AOM. There was no significant differences in microbiome diversity between NP and MEF samples (p = 0.31) (**Figure 1**). However, there appeared to be some difference at the level of individual OTUs. Specifically, *Veillonella* was reduced in the MEFs relative to NPs whereas *Alloiococcus otitidis* was increased (**Figure 2B** and **Table 2**).

### Comparison of Differences in Microbiome Diversities of the Samples Processed With Different Methods

We have six children whose MEF samples were stored in Trizol reagents before microbiome analysis and five children whose MEF samples were stored in PBS before microbiome analysis. We found that samples stored in PBS had significant greater diversities than those stored in Trizol (Shannon index, **Supplementary Figure 1**).

## Discussion

The pathogenesis, development, severity, and clinical outcomes of AOM are largely dependent on the resident composition of the NP microbiome and immune defense and few studies have provided an understanding of how the NP microbiome and molecular immune responses might be manipulated to favor the child host (Melendi et al., 2007; Alper et al., 2009; Wine and Alper 2012). The NP is the main ecological niche of AOM pathogens and is the site of transmission for otopathogens to others (contagion). Imbalance of the NP microbiome diversity (number and abundance) occurs during symptomatic infections (Pettigrew et al., 2012; Santee et al., 2016; Chonmaitree et al., 2017). Composition of the microbiome including the number of different species present (diversity), and the relative proportion of these species (evenness or abundance) are influenced by multiple factors (Lozupone and Knight 2008; Dominguez-Bello et al., 2010; Teo et al., 2015; Chonmaitree et al., 2017). In a study of 65 children with AOM and 74 children without AOM, Chonmaitree et al. (2017) have recently shown that viral URI frequency is positively associated with an increase in otopathogen colonization, and AOM frequency is associated with lower *Micrococcus* NP colonization. They also found during viral URI and AOM, increases in abundance in the NP of otopathogen genera when *Pseudomonas*, *Myroides*, *Yersinia*, and *Sphingomonas* are decreased. Finally, infant children with AOM in the first year were shown to have significant lower abundance of *Corynebacterium* and antibiotics significantly decrease *Corynebacterium* and *Dolosigranulum* (Pettigrew et al., 2012; Teo et al., 2015).

In our study, we found that NP microbiome diversity at onset of AOM significant lower compared with diversity during heath prior to AOM. The potential bacterial pathogens *Haemophilus*, *Moraxella*, and *Streptococcus* became the most abundant microbiota in the NP both during health prior to AOM and at onset of AOM. The commensal *Corynebacterium* was more abundant during health than at onset of AOM, although this difference did not reach statistical significance. Instead, *R. mucilaginosa*, *V. dispar*, *P. melaninogenica*, and certain species in the genus of *Streptococcus* appear to be less abundant in the NWs during AOM relative to health, suggesting these bacteria species may compete with the otopathogens for niche and their abundance reduces when the otopathogens prevail. Whether this is indeed the case has not been reported and may serve as new research directions in the microbiome field of AOM.

Relative to the difference between NWs of healthy children and children at onset of AOM, more OTUs diverged in their abundance between the MEFs of AOM children and NWs of healthy children. This increment in disparity suggests that after establishing in the NP, the invasion of otopathogens into ME and later growth in ME is associated with reduced abundance of commensal bacteria. This may be subtle and/or vary significantly among individuals, since very few OTUs differed significantly between the NWs and MEFs in children during AOM (**Figure 2** and **Table 2**).

The ME microbiome has been investigated recently in children with otitis media with effusion (OME), chronic otitis media, or recurrent AOM (Liu et al., 2011; Jervis-Bardy et al., 2015; Chan et al., 2016; Minami et al., 2017; Lappan et al., 2018). Several reports showed that the most abundant microbiome in ME of children with OME were *A. otitidis* followed by *Haemophilus*, *Moraxella*, and *Streptococcus* (Jervis-Bardy et al., 2015; Chan et al., 2016). We also observed a significant enrichment of *A. otitidis* in MEFs of AOM children, compared with NWs in AOM children or healthy children. It is unclear if *A. otitidis* is an otopathogen, a co-pathogen that facilitates biofilm formation, or a contaminant for the external ear canal skin flora. A prior study children with recurrent AOM found *Alloiococcus*, *Staphylococcus* sp. and *Turicella* were most abundant in the ME (Lappan et al., 2018), whereas adenoids microbiome was dominated by *H. influenzae*, *M. catarrhalis*, *S. pneumoniae*, *P. aeruginosa*, and *S. aureus* (Dirain et al., 2017). On the other hand, in chronic otitis media, Krueger et al. reported that the *Haemophilus* and *Moraxella* were the most abundant microbiota in the ME of children (Krueger et al., 2017), whereas Liu et al. reported that Pseudomonadaceae dominated in the ME, Streptococcaceae in the tonsil, and Pseudomonadaceae, Streptococcaceae, Fusobacteriaceae, and Pasteurellaceae dominated in the adenoid (Liu et al., 2011). In spite of the differences, our study and others suggest a resident microbiota in the ME that differs from NP after an initial ME infection has occurred.

Sample processing approaches and preservation methods may impact microbiome analysis results (Choo et al., 2015; Penington et al., 2018; Chen et al., 2019). In this study, we found that MEF samples stored in PBS had significant greater diversity of microbiome than MEF samples stored in the Trizol reagent after going through centrifugations.

Our study has limitations. Contamination during samples collection is always a concern for microbiome analysis. Contact with the external auditory canal during MEF samples collection may influence the accuracy in microbiome abundance of skin colonizers such as *Staphylococcus*, *Pseudomonas*, and *Alloiococcus*. (Johnston et al., 2019). The MEF samples were collected by tympanocentesis. Although we tired our best to avert contamination we cannot exclude the possibility contact of the external auditory canal by the tympanocentesis needle. Our participant cohort was small and sample size is a concern to interpret microbiome analysis results (Johnston et al., 2019). The 16S reads did not allow differentiation at the species level for most organisms identified; whole genome sequencing likely would have allowed better species level results. We expected to identify *Dolosigranulum pigrum* in some NP samples (Lappan et al., 2018). A recent report suggests *D. pigrum* may be mis-identified as *Alloiococcus* species during data analyses (Lappan et al., 2018).

In summary, in this study we found that the NP microbiome during health prior to AOM had greater diversity and are enriched in certain commensal bacteria, compared with the NP microbiome during AOM. The most abundant microbiota in the NP were known potential otopathogens (*Haemophilus*, *Moraxella*, and *Streptococcus*) along with nasal commensals such as *Corynebacterium.* At onset of AOM, no significant difference was found in microbiome diversity between NP and MEF. MEF may have a different microbiome profile than the NP suggesting a resident microbiota in the ME after a first ME infection. Sample processing and storage methods influence microbiome analysis results.

## Data Availability Statement

The raw data supporting the conclusions of this manuscript will be made available by the authors, without undue reservation, to any qualified researcher.

## Ethics Statement

The studies involving human participants were reviewed and approved by IRB of Rochester General Hospital. Written informed consent to participate in this study was provided by the participants' legal guardian/next of kin.

## Author Contributions

QX conceived the idea for the study, coordinated data collection, performed preliminary data analyses and prepared the initial draft of the manuscript. SG provided the resource for the completion of 16S rRNA sequence analyses. LX conducted final stages of data analyses and manuscript preparation. EG provided technical assistance throughout the project. MP provided overall guidance for the study and oversaw its completion.

## Conflict of Interest

The authors declare that the research was conducted in the absence of any commercial or financial relationships that could be construed as a potential conflict of interest.

## Supplementary Material

Supplementary Figure 1 Sample processing approach affects microbiota diversity. MEF samples were either stored in PBS or in Trizol after centrifugation. Both types of samples were analyzed by 16S rRNA sequencing. Shannon diversity index was calculated and compared between the two sets of samples by one tailed t test.
