# Supplementary figures and images for: Comparative Analysis of Microbiome in Nasopharynx and Middle Ear in Young Children With Acute Otitis Media
Source: Front Genet. 2019 Nov 19;10:1176. doi: 10.3389/fgene.2019.01176 (PMC6877732; doi:10.3389/fgene.2019.01176)

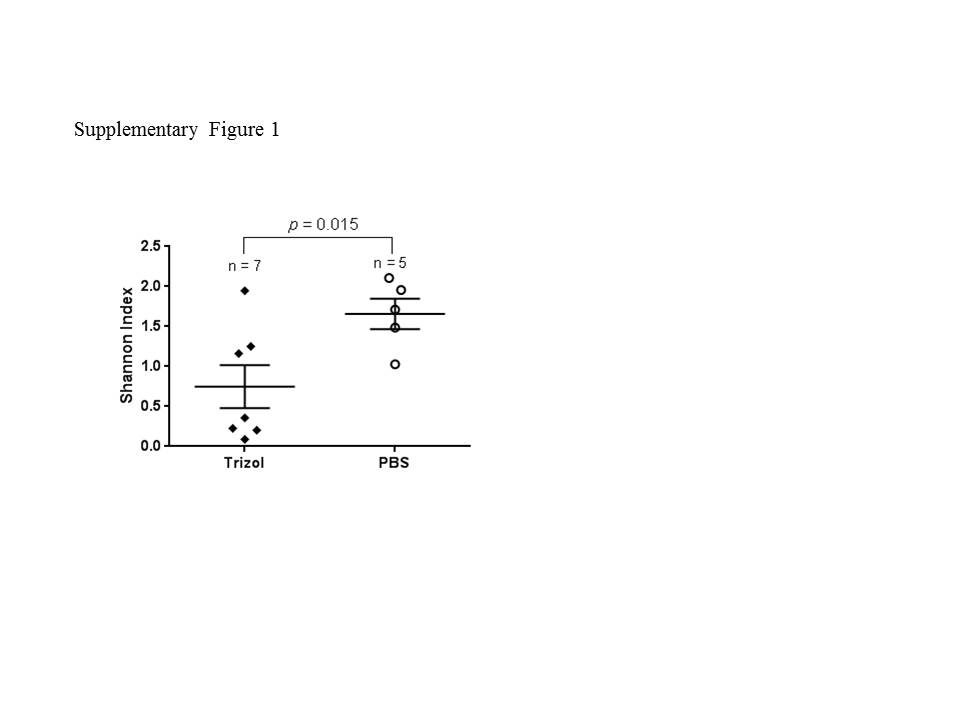

Supplement: Supplementary Figure 1 — Sample processing approach affects microbiota diversity. MEF samples were either stored in PBS or in Trizol after centrifugation. Both types of samples were analyzed by 16S rRNA sequencing. Shannon diversity index was calculated and compared between the two sets of samples by one tailed t test. [file Image_1.jpeg]
